# Dendritic Spines and Pre-Synaptic Boutons Are Stable Despite Local Deep Hypothermic Challenge and Re-Warming In Vivo

**DOI:** 10.1371/journal.pone.0036305

**Published:** 2012-05-01

**Authors:** Yicheng Xie, Shangbin Chen, Timothy Murphy

**Affiliations:** 1 Kinsmen Laboratory of Neurological Research, Department of Psychiatry, University of British Columbia, Vancouver, British Columbia, Canada; 2 Brain Research Centre, University of British Columbia, Vancouver, British Columbia, Canada; University of Nebraska Medical Center, United States of America

## Abstract

**Background and Purpose:**

Deep hypothermia to 20°C is used clinically for major pediatric and adult surgical procedures. In particular, it is used in the “standstill operation" where blood flow is stopped for up to 30 min. Patients recovering from these procedures can exhibit neurological deficits. Such deficits could arise from changes to dendritic spines and plasticity-induced changes in network function as a result of cooling and/or re-warming. In the brain, each dendritic spine represents a single excitatory synapse and their number can be reflective of injury or plasticity-induced changes in network function. This research sought to determine whether deep hypothermia and re-warming have detrimental effects on synaptic stability and network function.

**Methods:**

In vivo 2-photon (2-P) imaging in green/yellow fluorescent protein (GFP/YFP)-expressing transgenic mice was performed to determine whether 4 hours of deep hypothermia and 2 hours of re-warming can have relatively covert effects on dendritic spine and presynaptic bouton stability. At the same time, electroencephalographic (EEG) activity was recorded to evaluate network function during deep hypothermia and re-warming.

**Results:**

We report that deep hypothermia and subsequent re-warming did not change the stability of dendritic spines or presynaptic boutons in mouse somatosensory cortex measured over 8 hours. As expected, deep hypothermia attenuated ongoing EEG activity over 0.1–80 Hz frequencies. The effects on EEG activity were fully reversible following re-warming.

**Conclusion:**

These results are consistent with deep hypothermia being a safe treatment which could be applied clinically to those undergoing major elective surgical procedures.

## Introduction

Hypothermia is the most widely used neuroprotective intervention in stroke and other brain injuries [1], [2], [3]. Various depths of hypothermia, including mild (∼32°C), moderate (∼28°C) and deep (∼22°C), have been applied to protect the brain from different cerebral injuries and consequences of cardiac surgery [4], [5]. Of particular concern is deep hypothermia since cooling could potentially alter cytoskeletal dynamics [6], leading to changes in synaptic function [7]. Clinical reports have also shown that approximately 90 mins of deep hypothermia (21±3°C) has several associated neurological risks during cardiopulmonary bypass in humans [8]. Deep hypothermia can induce neurological complications such as middle cerebral artery stroke, temporal lobe hematoma, cerebral edema, and hemorrhage [3]. However, whether these complications are due to the direct effects of deep hypothermia on brain tissue is unclear. Furthermore, for many patients deep hypothermia is produced for only extreme circumstances during no-flow standstill operations to repair brain aneurysms or aortic arch defects [3], [9] making controlled studies impossible. Therefore, systemic investigations of deep hypothermia's effects are required for guiding its use in surgical procedures. Interestingly, body temperature, arterial blood pressure, and heart rate are unchanged over hours under local cortical hypothermia (cortical surface temperature at 26°C) in rodents [10]. These studies suggest that it may be possible to directly regulate cortical temperature without major systemic side-effects. In this study, using transgenic mice in which axons, dendrites, and spine synapses are labeled with GFP or YFP [11], [12], [13] and a custom-made headplate for local surface cortical cooling and subsequent re-warming [14], we have examined the turnover of individual pre and postsynaptic structural elements *in vivo* under acute deep hypothermia (∼4 h) and re-warming (∼2 h) using *in vivo* 2-photon imaging [15]. Although analysis of dendritic spines could be made using histological specimens, relatively low percentage “covert" changes in spine number could be overlooked when different groups of neurons and animals are compared. We have longitudinally examined the stability of the same dendritic spines or pre-synaptic boutons, combined with EEG recording during deep hypothermia and re-warming.

## Results

**Figure 1:**
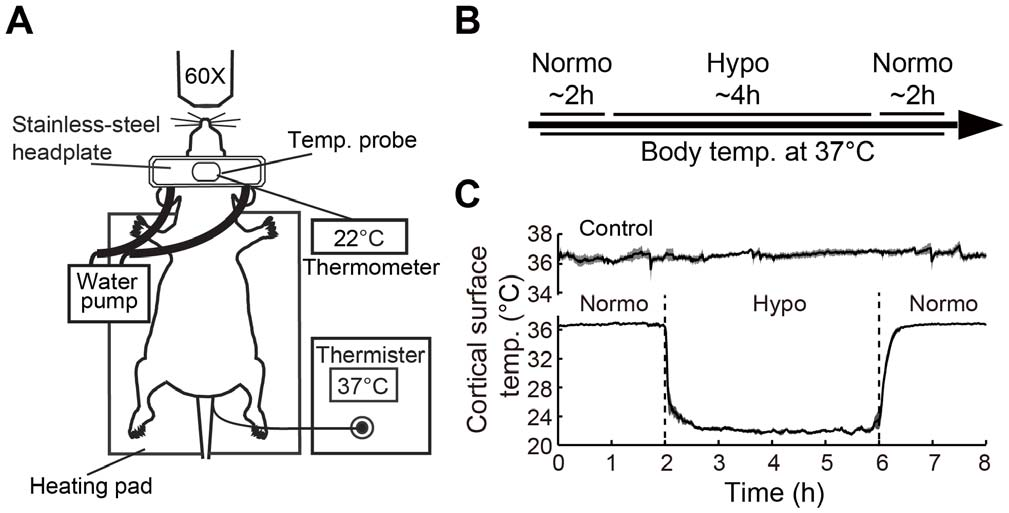
Overview of experimental set-up and temperature regulation. A, Experimental set-up for combined two-photon (2-P) imaging and temperature regulation. A pump circulates water through a custom-designed head-plate to induce hypothermia or normothermia while body temperature is maintained at 37°C. Surface temperature was recorded by a temperature probe. B, Experimental timeline, ∼2 h normothermia (∼36.5°C) for baseline recording and imaging, 4 h of deep hypothermia (∼22°C) and 2 h of re-warming (∼36.5°C). C, Temperature recording over 8 hours for both control group (n = 4) and deep hypothermia combined with re-warming group (n = 7). Data represent mean ± SEM.

In this study, we investigated the effects of local deep hypothermia and re-warming on dendritic spine and presynaptic bouton structure and neuronal activity as measured by EEG. A custom-made stainless steel head-plate adhered to the skull was connected with tubing to a cooling pump, and used to alter cortical surface temperature (Fig. 1A). Temperature probe recordings verified that the area of cortical surface being thermally regulated was maintained at ∼22°C during hypothermia and at ∼36.5°C during baseline and re-warming (Fig. 1B). *In vivo* 2-P imaging of GFP/YFP expressing layer 5 apical dendritic and axonal structures (e.g. dendrites, spines and boutons) was used to assess hypothermia- or re-warming-induced dendritic and axonal structural changes.

**Figure 2:**
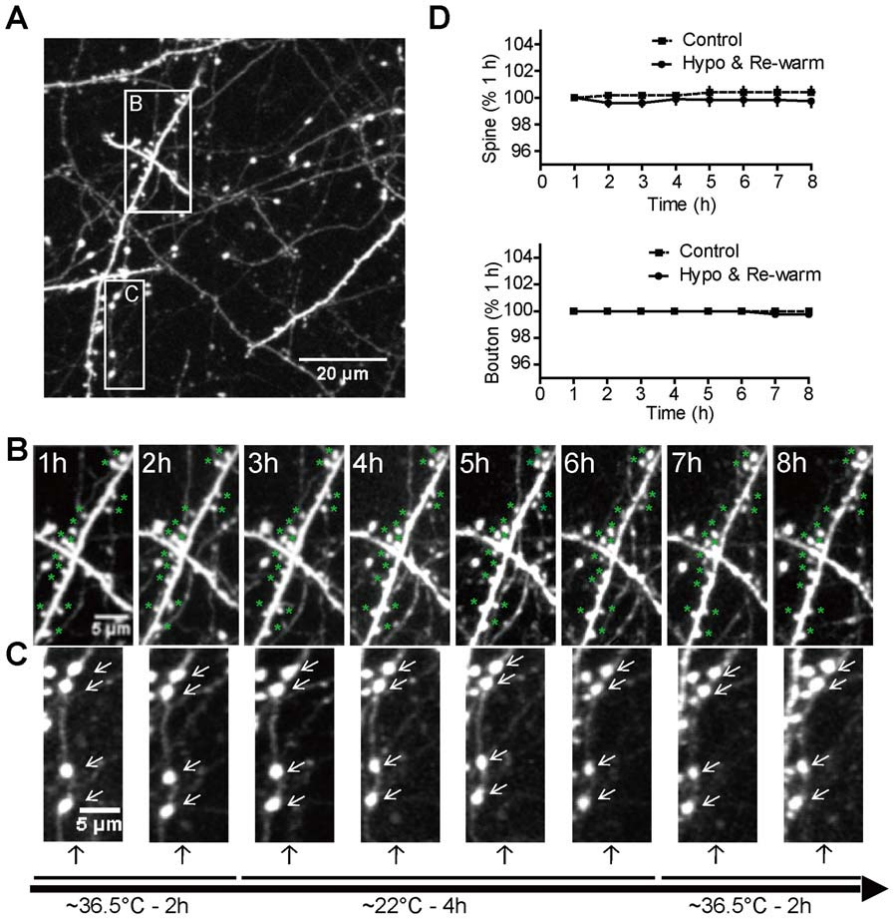
Local deep hypothermia and re-warming does not induce significant changes in boutons or dendritic spines. A, Maximal intensity z- projection of 30 planar images (taken 1 µm apart) at the 1 h time point after induction of normothermia depicting YFP/GFP labeled dendrites. B, Time-lapse images showing post-synaptic dendritic spines. “*" indicates an individual spine. C, Time-lapse images showing pre-synaptic boutons. Arrow indicates an individual bouton. D, Statistical analysis of spine and bouton numbers (4 mice for control group, 7 mice for hypothermia combining re-warming group) over 8 h. Data represent mean ± SEM. The number of boutons or spines was compared to the 1 hour time point after induction of normothermia. No statistically significant difference was found by one-way ANOVA followed by Bonferroni's post-tests.

Under 2-P microscopy, dendritic and axonal structures were monitored with a 60× objective (Fig. 2A). We observed that dendrites were intact during deep hypothermia and following re-warming and no apparent changes in morphology were observed such as blebbing (Fig. 2B) that can accompany ischemia [16], [17], [18]. Closer longitudinal examination revealed that both spines (Fig. 2B) and presynaptic boutons (Fig. 2C) were stable. No statistically significant differences in spine and bouton numbers (p-value>0.05, n = 7) were observed by one-way ANOVA in either deep hypothermia or following re-warming, compared to baseline imaging (image acquired at 1 h time point), which was similar to an control group (n = 4) maintained at normothermia over 8 h (Fig. 2D). In 933 spines monitored from 7 mice, we observed 6 spines were lost (0.6%) and 2 spines were gained (0.2%) after experiencing 4 h of deep hypothermia and following 2 h of re-warming. Similarly, in 630 boutons monitored from these mice, 2 boutons were lost (0.3%) and no boutons were gained (0%) in the same experiment. In the control group treated with normothermia (4 mice), a total of 330 spines and 145 boutons were monitored and analyzed over 8 h. Addition of one spine (0.3%) was observed and no change of bouton number (0%) was detected.

**Figure 3:**
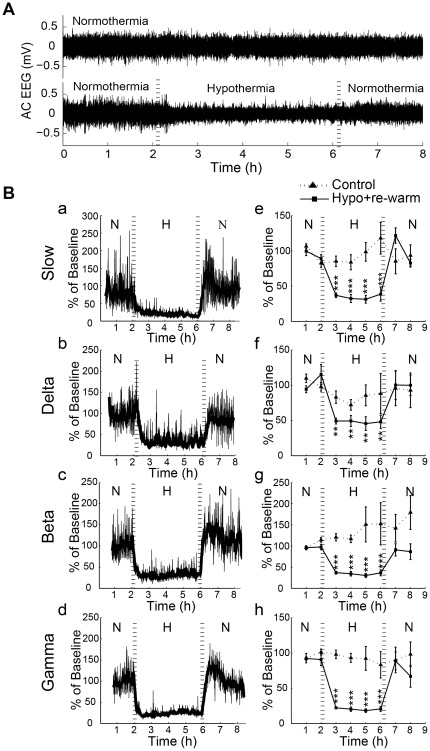
Local deep hypothermia reversibly attenuates ongoing EEG activity over 0.1–80 Hz frequencies. A, Representative filtered EEG traces (0.1–80 Hz) from an animal with deep hypothermia combined with re-warming. B, Deep hypothermia reversibly attenuated EEG power at different frequency bands. Representative (a–d) and mean (e–h) EEG power over 8 h from control group (4 mice) or deep hypothermia combined with re-warming group (7 mice) for low (0.1–1 Hz, a,e), delta (1–3 Hz, b,f), beta (12–30 Hz, c,g), and gamma (30–80 Hz, d,h) frequency bands. N: normothermia (∼36.5°C), H: hypothermia (∼22°C). Data represent mean ± SEM. e–h, all mean values of power were compared to those of the 1 h time point during normothermia, **p<0.01, ***p<0.001, one-way ANOVA followed by Bonferroni's post-tests.

Although relatively few changes in dendritic or axonal structures were observed, in parallel we investigated whether neuronal activity would also change during deep hypothermia and following re-warming by recording spontaneous EEG. After filtering EEG data in the frequency range of 0.1∼80 Hz, we observed that under isoflurane anesthesia in normothermic conditions that spontaneous EEG activity was stable through 8 h of recording, whereas deep hypothermia decreased EEG amplitude, which recovered to baseline following re-warming (Fig. 3A). Power spectrum analysis of 4 frequency bands was performed during deep hypothermia and re-warming; including slow (0.1–1 Hz), delta (1–3 Hz), beta (12–30 Hz), and gamma (30–80 Hz). In all 4 frequency bands, we found a decrease in power during deep hypothermia and almost full recovery in power following re-warming (Fig. 3Ba–d). Statistical analysis showed that in all 4 frequency bands, deep hypothermia decreased power to 50% or less at the 1 h time point (p-values<0.01 or 0.001, n = 7) (Fig. 3Be–h). During re-warming, the power was restored to approximately 90∼100% of that recorded during the beginning of normothermia for slow, delta, and beta frequency bands (p-value>0.05, n = 7) (Fig. 3Be–g). There was a trend towards a decrease in gamma power after re-warming (89%±13% and 67%±16% for the 1 and 2 h time points, respectively), but no significant difference was observed from the 1 h experiment point (p-value>0.05, n = 7) (Fig. 3Bh). Under isoflurane anesthesia, normothermia did not induce statistically significant changes in power of the same frequency bands over 8 h of recording (Fig. 3Be–f). Therefore, we conclude that deep hypothermia reversibly attenuated ongoing EEG activity over different frequency bands, and the effect on brain electrical activity was reversible with re-warming.

## Discussion

In the present study, we used *in vivo* 2-P imaging of individual pre and post-synaptic components to investigate the effects of deep hypothermia on neuronal structure. We employed local control of brain temperature during longitudinal 2-P imaging *in vivo*. Using this method, we can control brain surface temperature from 22°C to 36.5°C. At the same time, mouse body temperature was maintained at ∼37°C using a feedback heating pad system to avoid potentially indirect effects. In this study, the rate of induction of re-warming was relatively fast, compared to human hypothermic treatment (0.1°C to 0.2°C per hour) [19]. We report that deep hypothermia and re-warming did not change the stability of neuronal structure, including dendrites, their spines and boutons in the somatosensory cortex of mice. EEG recording showed that deep hypothermia reversibly attenuated spontaneous neuronal activity in all slow, delta, beta and gamma frequency bands.

Hypothermia has been applied in treating variety of CNS diseases [4]. However, systemic deep hypothermia (15–21°C, 17–52 mins) has several associated risks with respect to neurological complications in patients [3]. In addition, 48 h of systemic deep hypothermia can induce severe side-effects in monkey and cat such as a decrease in cerebral blood flow and energy metabolites, and can even lead to death. But these effects may be due to systemic deficits, such as a decrease in heart rate and blood pressure [20]. Experimentally, 6 h of deep hypothermia (<24°C) can significantly reduce infarct volumes in rodents [21]. Recently, we have reported that moderate or deep local hypothermia can delay the onset of ischemia-induced dendritic damage and promote recovery after reperfusion [14]. However, the effect of prolonged deep local hypothermia itself on neuronal structure (in particular pre-synaptic elements) was not directly investigated in this study, nor was the effect of re-warming. GFP-M and YFP-H mice were used in this study. Based on their expression pattern, dendrites and spines from cortical layer 5 neurons [11], as well as axons and boutons from thalamocortical projection neurons and superficial pyramidal neurons [22] were imaged. Our data suggest that 4 h of deep hypothermia to ∼22°C does not significantly affect stability of neuronal structures, including dendrites, spines, and presynaptic boutons. Also, re-warming, at a rapid rate compared to clinical applications [19], did not affect the stability of neuronal structures. The turnover ratio of spines during treatment with 4 h of deep hypothermia and following 2 h re-warming under isoflurane anesthesia (∼0.8%) was similar to that reported previously over 6 h of urethane anesthesia (∼0.5%) [23]. We also found that the turnover ratio of boutons was similar to results reported under normal conditions [24]. Interestingly, in contrast to the findings *in vitro*, we did not observe dendritic blebbing during chilling, nor did we observe proliferation of spines during re-warming [6]. Perhaps these differences are because these *in vitro* effects were observed at even lower temperatures (5–7°C). Consistent with lower temperatures having larger effects on spines, findings in ground squirrels show decreases in the CA3 pyramidal neuron dendritic spines in the middle of hibernation and excessive proliferation after arousal from hibernation [25], [26]. Further study is required to investigate dendritic spine dynamics at lower temperatures (e.g., 6°C) and over longer time points. Perhaps these studies will be aided by less invasive imaging preparations such as reinforced thin skull preparations [27], [28].

During deep hypothermia, EEG activity was depressed substantiating the hypothesis that reduced cerebral metabolic rate is a mechanism of the neuroprotective effects of hypothermia in some cardiac surgical procedures [4]. Following re-warming, the EEG recovered and was maintained for at least 2 h. The recovery of activity following re-warming contrasts with results found following even brief global ischemia where neuronal activity was suppressed for hours despite full reperfusion [29], [30]. Notably, the rate of re-warming in our experiment was relatively fast which may cause a temporary mismatch between cerebral metabolic rate and cerebral blood supply [31]. Since neuronal structure was intact during re-warming, we suggest that accelerated re-warming after local hypothermia may not be detrimental at least to the structures we have monitored. Given that previous work indicates that cortical neuronal death may appear 1 week after deep hypothermic circulatory arrest (90 mins) in newborn pigs [32], we concede that prolonged monitoring following re-warming would be an advantage for future work. Unfortunately, the invasive nature of the cooling system has limited our work to acute experiments.

In conclusion, we have found that local deep hypothermia and re-warming does not acutely alter the stability of neuronal synaptic structure in mice. These results suggest that deep hypothermia is a safe treatment with respect to synaptic networks which could be applied clinically to those undergoing elective cardiac surgical procedures.

## Materials and Methods

### Animal, surgical procedures and regulation of cortical temperature

Adult male C57BL/6 mice (8∼10 weeks of age) expressing yellow fluorescent (YFP)-H or green fluorescent protein (GFP)-M [11] were used. Experimental protocols were approved by the University of British Columbia animal care committee and conformed to the Canadian Council on Animal Care and Use guidelines. Body temperature was maintained throughout surgical and imaging procedures at 37±0.5°C. In all animals, cortical surface temperature was measured using a thermocouple (IT-24P, Physitemp Instruments Inc, Clifton, NJ) placed over the cortical surface (estimated to be less than 0.5 mm from the surface) within agarose. The cortical surface temperature was maintained at 22°C by attaching a custom-made stainless steel head plate [14] as described in Fig. 1A. Since temperature measurements were made from the surface it is possible that cortical temperature maybe greater within the vicinity of local blood flow.

### In vivo 2-photon imaging

To mimic clinical conditions, isoflurane was chosen as the anesthetic and was used at 1.5% in air [14]. The method of imaging has been described before [16], [23], [30] as is based on an adaptation of procedures first described for rats [33], [34]. Briefly, mice underwent surgical procedures to perform a 3×3 mm craniotomy over the somatosensory cortex. 2-P imaging of apical dendrites of layer 5 neurons was performed. The imaging was restricted to the first 40 µm of cortex within the hindlimb somatosensory cortex border as determined by mouse atlas coordinates [35]. To reduce photon and photomultiplier tube noise, a median filter (radius, 1 pixel) was applied to all images.

### EEG recording

For EEG recording, a Teflon coated silver wire (0.125 mm; World Precision Instruments, Sarasota, FL) was placed on the surface of the cortex within the agarose. A reference electrode was placed on the nasal bone under the skin. The cortical signal was amplified and filtered (0.1–1000 Hz) using a differential alternating current (AC) amplifier (Model 1700, A-M Systems) and digitized (1000 Hz) using a 1322A Digidata (Molecular Devices, Sunnyvale, CA). Additional off-line filtering was also routinely performed. EEG data were collected using AxoScope 10 and analyzed using Clampfit 10 (Molecular Dynamics).

### Image and statistical analyses

Dendritic spines and axonal boutons were counted in the same sections of cortex using blinded manual analysis. The mean power of each spectrum before induction of deep hypothermia was set as the baseline (100%). All the power spectrums relative to percentage of baseline were filtered using 20 time points zero-phase digital filtering and aligned to a starting time point (130 mins) of deep hypothermia using Matlab2010. Temperature data were recorded each minute. A one-way ANOVA followed by Bonferroni's post-tests were used to compare differences in dendritic spines, post-synaptic boutons, and EEG power spectrums of each time point to 1 h time point in experimental groups. Significance was set at p<0.05.
